# A Comprehensive Analysis of the Association Between the EORTC QLQ‐C30 Questionnaire and Cachexia in Patients With Gastric Cancer

**DOI:** 10.1002/jcsm.13859

**Published:** 2025-06-12

**Authors:** Xi Zhang, Xiang‐Ting Dai, Chao Wang, Jia‐Xin Huang, Ping‐ping Jia, Meng Tang, Chun‐Hua Song, Wei Li, Han‐Ping Shi, Ming‐Hua Cong

**Affiliations:** ^1^ Department of Comprehensive Oncology National Cancer Center/National Clinical Research Center for Cancer/Cancer Hospital, Chinese Academy of Medical Sciences and Peking Union Medical College Beijing China; ^2^ Key Laboratory of Cancer FSMP for State Market Regulation Beijing China; ^3^ Beijing International Science and Technology Cooperation Base for Cancer Metabolism and Nutrition Beijing China; ^4^ Department of Clinical Nutrition Beijing Shijitan Hospital, Capital Medical University Beijing China; ^5^ Department of Epidemiology College of Public Health, Zhengzhou University Zhengzhou China; ^6^ Cancer Center of the First Hospital of Jilin University Changchun China; ^7^ Department of Gastrointestinal Surgery Beijing Shijitan Hospital, Capital Medical University Beijing China

**Keywords:** cachexia, EORTC QLQ‐C30, gastric cancer, prognosis, quality of life, survival

## Abstract

**Background:**

Cancer cachexia is associated with poor quality of life (QoL) and reduced survival in patients with cancer. The European Organization for Research and Treatment of Cancer (EORTC) QLQ‐C30 is a widely used cancer‐specific health‐related QoL questionnaire that comprises 15 scales, consisting of five multi‐item functional scales, three multi‐item symptom scales, six single‐item symptom scales and a global health and quality‐of‐life scale. Our study aimed to analyse the association of each scale in the EORTC QLQ‐C30 questionnaire with cachexia and explore its influence on survival outcomes in patients with gastric cancer and cachexia.

**Methods:**

This multicentre cohort study enrolled 3158 patients with gastric cancer, among whom 1711 were diagnosed with cachexia. Logistic regression analysis was conducted to identify the individual scales of the EORTC QLQ‐C30 questionnaire significantly affected by cachexia. The Cox model was employed to evaluate the prognostic performance of EORTC QLQ‐C30 questionnaire scales in patients with gastric cancer and cachexia.

**Results:**

In this study, the median age of patients with gastric cancer was 67.00 (interquartile range [IQR], 58.00–74.00) years, with 2178 (69.0%) men and 980 (31.0%) women. In logistic regression analyses, scales such as physical function (*p* < 0.001), global quality of life (*p* = 0.022), fatigue (*p* < 0.001), nausea and vomiting (*p* < 0.001), dyspnoea (*p* = 0.004), insomnia (*p* = 0.007), loss of appetite (*p* < 0.001), constipation (*p* < 0.001), diarrhoea (*p* = 0.017) and summary score (*p* = 0.039) were significantly associated with cachexia in patients with gastric cancer. According to the receiver operating characteristics (ROC) curves, loss of appetite was the most significant scale associated with cachexia. Based on multivariate Cox analyses, the scales of physical function (HR = 1.33, 95% CI = 1.00–1.77, *p* = 0.049), role function (HR = 1.48, 95% CI = 1.15–1.90, *p* = 0.002), social function (HR = 1.90, 95% CI = 1.40–2.56, *p* < 0.001), global quality of life (HR = 1.45, 95% CI = 1.05–2.00, *p* = 0.026), financial impact (HR = 1.53, 95% CI = 1.15–2.03, *p* = 0.003) and summary score (HR = 1.39, 95% CI = 1.01–1.91, *p* = 0.042) were independent risk factors for survival in patients with gastric cancer and cachexia. The concordance index (C‐index) and area under the curve (AUC) value for survival prediction were the highest for the social function scale.

**Conclusion:**

The QoL of gastric cancer patients with cachexia was significantly reduced. Certain scales in the EORTC QLQ‐C30 were significantly associated with cachexia, especially the loss of appetite scale, and survival outcomes in patients with gastric cancer, especially social function. Emphasizing these scales can heighten our awareness of the impact of cachexia on QoL and enhance our ability to predict the survival of patients with gastric cancer and cachexia.

**Trial Registration:** ChiCTR: 1800020329

## 1 Introduction

Gastric cancer, a major global health concern, is the fifth most common cancer and the fourth most common cause of cancer death, with approximately 769 000 deaths globally reported in 2020 [1]. Additionally, it is a leading contributor to the global cancer burden, with a significant impact quantified through the metric of disability‐adjusted life years lost [2]. An alarming rise in gastric cancer cases persists in certain countries, regions and within‐specific demographic groups. Furthermore, clinicians anticipate an increase in gastric cancer cases due to aging populations, underscoring the urgent need for targeted interventions and research [3]. Data from palliative care settings indicate that cachexia prevalence remain consistently high at the end of life, regardless of cancer type [4]. Gastric cancer, often diagnosed at an advanced stage, is among the cancers most commonly associated with cachexia [5].

Cachexia, a prevalent complication of cancer with multifactorial origins, is characterized by tissue wasting, weight loss (mainly lean body mass), elevated resting energy expenditure and metabolic disturbances [6]. It also involves ongoing skeletal muscle wasting (either with or without fat mass depletion) that is not readily reversible through standard nutritional interventions and results in increasing functional decline [7]. The established diagnostic criteria for cachexia include weight loss exceeding 5%, or a reduction of more than 2% in those manifesting depletion, as indicated by their body mass index (BMI below 20 kg/m^2^) or reduced skeletal muscle mass (sarcopenia) [7].

It is estimated that cachexia impacts up to 80% of cancer patients, and contributes to 20–40% of all cancer‐related deaths [8]. Patients with cachexia frequently experience anorexia, resulting in decreased food intake and elevated fatigue [9]. Furthermore, cancer patients diagnosed with cachexia encounter prolonged hospital stays, higher medical expenses and exacerbated functional impairment compared with those without cachexia [10]. Cachexia has caused substantial psychological distress among patients, their families and caregivers [11]. All of these collectively have a detrimental impact on patients' QoL [7]. Moreover, when patients with gastric cancer develop cachexia, their survival rate is reduced. This suggests that cachexia simultaneously impacts the QoL and prognosis of patients with gastric cancer. Therefore, identifying the factors most affected by cachexia is crucial for mitigating the potential harm of cachexia and extending the survival time of patients with cancer.

Studies have highlighted the importance of assessing cachexia patients' QoL, given that current treatments for cachexia are primarily palliative, and efficacy should be assessed using criteria beyond survival alone [12]. The EORTC QLQ‐C30 was developed as the second‐generation questionnaire by the EORTC study group in 1986 to evaluate the QoL of patients participating in international clinical trials [13]. It is a widely recognized and applied tool for QoL assessment worldwide, especially in evaluating the QoL of patients with cancer [13]. Although the QLQ‐CAX24 (which supplements the QLQ‐C30) and the FACIT‐FAACT can be used to assess QoL in patients with cachexia, the QLQ‐C30 was selected due to its most widespread adoption in clinical research evaluating cancer patients with cachexia. As noted in a recent systematic review, the QLQ‐C30 remains the most frequently used QoL measure in cachexia trials (60%), followed by different FACIT questionnaires (34%) [14].

Given that cachexia impacts the QoL, identifying EORTC QLQ‐C30 scales most affected by cachexia can enhance the assessment of patients' QoL and subsequently predict survival outcomes in patients with cancer. However, there is no detailed research on the relationship between cachexia and each EORTC QLQ‐C30 scale. Our study aims to identify independent scales within the EORTC QLQ‐C30 that are significantly influenced by cachexia in patients with gastric cancer. Additionally, it seeks to evaluate the association between the EORTC QLQ‐C30 scales and overall survival (OS) in patients with gastric cancer and cachexia.

## 2 Patients and Methods

### 2.1 Study Population and Design

Figure S1 is the main flow chart of this study. This multicentre prospective cohort study consisted of patients diagnosed with cancer between 2013 and 2022 from the Investigation on Nutrition Status and Clinical Outcomes of Common Cancer (INSCOC) project of China [15]. Patients diagnosed with gastric cancer were included. The exclusion criteria were (a) hospital stay of less than 48 h, (b) incomplete EORTC QLQ‐C30 questionnaire data and (c) lack of essential baseline data for analysis.

### 2.2 Baseline Characteristics

Data collected during hospital stays by specialized personnel included patients' baseline profiles such as age, gender, comorbidities (diabetes and hypertension), coronary heart disease, smoking status, alcohol consumption, tumour/node/metastasis (TNM) stage and anticancer treatments including surgery, chemotherapy and radiotherapy. Additionally, Nutrition Risk Screening (NRS) 2002 scores, Eastern Cooperative Oncology Group (ECOG) performance status (PS), patient‐generated subjective global assessment (PG‐SGA), BMI, calf circumference (CC), handgrip strength (HGS), albumin level, complete blood count including white blood cell, neutrophil, lymphocyte, platelet counts and neutrophil‐to‐lymphocyte ratio (NLR) were assessed. Pathological staging was performed according to the 8th edition of the American Joint Committee on Cancer TNM staging system. Patient performance was evaluated using the ECOG grade, which was converted by using Karnofsky performance status (KPS) as follows: ECOG‐PS 0 (KPS 100), ECOG‐PS 1 (KPS 90 to 80), ECOG‐PS 2 (KPS 70 to 60), ECOG‐PS 3 (KPS 50 to 40) and ECOG‐PS 4 (KPS 30 to 0) [16]. Patient nutritional status was assessed based on two scoring systems: an NRS 2002 score ≥ 3 indicated risk of malnutrition and a PG‐SGA score ≥ 4 identified malnutrition [17]. BMI (kg/m^2^) was calculated by dividing patient weight in kilograms by the square of height in meters. HGS was measured using an electronic handgrip dynamometer with the patient's nondominant hand. CC was measured with the patient in the supine position with knees flexed at 90°.

### 2.3 EORTC QLQ‐C30 Questionnaire

The QoL was assessed using the EORTC QLQ‐C30 Questionnaire, version 3.0. This questionnaire includes five multi‐item functional scales, three multi‐item symptom scales, six single‐item symptom scales and a two‐item global quality‐of‐life scale. Each item was assessed on a 0‐ to 100‐point scale. The summary score of the EORTC QLQ‐C30 was calculated based on the formula provided: summary score = ([physical functioning + role functioning + social functioning + emotional functioning + cognitive functioning] + [100 − fatigue] + [100 − pain] + [100 − nausea and vomiting] + [100 − dyspnoea] + [100 − insomnia] + [100 − appetite loss] + [100 − constipation] + [100 − diarrhoea])/13 [18].

### 2.4 Outcomes of Study

Cachexia and OS were the primary outcomes measured in our study. According to an international consensus [7], cancer cachexia is defined and classified as follows: (1) weight loss > 5% over past 6 months (in the absence of simple starvation); (2) BMI < 20 and any degree of weight loss > 2%; (3) an appendicular skeletal muscle index consistent with sarcopenia (men < 7.26 kg/m^2^; women < 5.45 kg/m^2^) and any degree of weight loss > 2%. Skeletal muscle depletion was measured by mid‐upper arm muscle area using anthropometry (men < 32 cm^2^ and women < 18 cm^2^). Overall survival was defined as the number of months a patient survived between the date of study enrolment and the date of death. Any patients lost to follow up or still alive at the time of evaluation are censored.

### 2.5 Statistical Analysis

We conducted statistical analyses using R software, version 4.3.1 (The R Foundation; https://www.r‐project.org/). Continuous variables were transformed into categorical variables based on recognized cut‐off values (e.g., for age and BMI) or expressed as median number interquartile range (IQR) and compared using the Mann–Whitney *U* test. For the variables HGS and CC, patients were categorized into two groups according to the 15th percentile of their distribution in the study population. For the scores of the 15 scales and the summary score of the EORTC QLQ‐C30, patients were categorized into two groups by quartiles. Categorical variables were compared using *χ* ^2^ or Fisher's exact tests and presented as absolute numbers or percentages. Univariate and multivariate logistic regression analyses were used to investigate independent variables significantly associated with cachexia, reporting odds ratios (ORs) with 95% confidence intervals (CIs). Independent predictors of poor OS were identified using hazard ratios (HRs) and 95% CIs based on Cox regression analyses. The predictive value was assessed using the C‐index and ROC curves. The Kaplan–Meier method, as determined by the log‐rank test, was used to assess OS prediction performance. The C‐index values with 95% CIs were compared using the Mann–Whitney *U* test. Statistical testing was two‐sided, with *p* < 0.05 considered statistically significant.

## 3 Results

### 3.1 Patient Baseline Characteristics

Our study enrolled a total of 3158 patients diagnosed with gastric cancer in pathology from the multicentre database. Patient baseline characteristics, including demographic information, tumour‐related characteristics and laboratory data stratified by cachexia, are presented in Table 1. Cachexia is more prevalent in women with gastric cancer (57.1%) compared with men (52.8%). In comparison with the early stage of gastric cancer, where the prevalence of cachexia was 49.4%, the prevalence was significantly higher in patients with advanced gastric cancer, at 56.7%. Patients with cachexia had a higher proportion of individuals without a history of surgery (59.4% vs. 48.1%), a greater proportion with a history of radiotherapy (77.5% vs. 59.4%) and a higher proportion with a history of chemotherapy (59.7% vs. 50.3%). Additionally, a higher percentage of patients with cachexia had NRS 2002 scores of 3 or higher (70.3% vs. 18.6%), ECOG grades above 1 (63.7% vs. 52.1%), had lower values of BMI, CC, HGS, albumin, WBC and lymphocyte count and showed higher values in NLR.

**TABLE 1:** Baseline characteristics stratified by cachexia in gastric cancer patients.

Variables	All patients (*n* = 3158)	Without cachexia (*n* = 1447)	With cachexia (*n* = 1711)	*p*
Age, years	67.00 (58.00–74.00)	67.00 (58.00–73.00)	67.00 (58.00–74.00)	0.800
Gender (male/female)	2178/980 (69.0%/31.0%)	1027/420 (71.0%/29.0%)	1151/560 (67.3%/32.7%)	0.028*
Diabetes (yes/no)	213/2945 (6.7%/93.3%)	105/1342 (7.3%/92.7%)	108/1603 (6.3%/93.7%)	0.326
Hypertension (yes/no)	491/2667 (15.5%/84.5%)	219/1228 (15.1%/84.9%)	272/1439 (15.9%/84.1%)	0.589
CHD (yes/no)	99/3059 (3.1%/96.9%)	47/1400 (3.2%/96.8%)	52/1659 (3.0%/97.0%)	0.816
Smoking (yes/no)	1416/1742 (44.8%/55.2%)	662/785 (45.7%/54.3%)	754/957 (44.1%/55.9%)	0.362
Drinking (yes/no)	699/2459 (22.1%/77.9%)	324/1123 (22.4%/77.6%)	375/1336 (21.9%/78.1%)	0.782
TNM stages				< 0.001*
I	427 (13.5%)	220 (15.2%)	207 (12.1%)	
II	661 (20.9%)	331 (22.9%)	330 (19.3%)	
III	1339 (42.4%)	608 (42.0%)	731 (42.7%)	
IV	731 (23.1%)	288 (19.9%)	443 (25.9%)	
Surgery (yes/no)	1465/1693 (46.4%/53.6%)	760/687 (52.5%/47.5%)	705/1006 (41.2%/58.8%)	< 0.001*
Radiotherapy (yes/no)	89/3069 (2.8%/97.2%)	20/1427 (1.4%/98.6%)	69/1642 (4.0%/96.0%)	< 0.001*
Chemotherapy (yes/no)	1299/1859 (41.1%/58.9%)	524/923 (36.2%/63.8%)	775/936 (45.3%/54.7%)	< 0.001*
NRS2002 (< 3/≥ 3)	983/2175 (31.1%/68.9%)	800/647 (55.3%/44.7%)	183/1528 (10.7%/89.3%)	< 0.001*
ECOG grade (≤ 1/> 1)	2594/564 (82.1%/17.9%)	1242/205 (85.8%/14.2%)	1352/359 (79.0%/21.0%)	< 0.001*
PG‐SGA (< 4/≥ 4)	1888/1270 (59.8%/40.2%)	1234/213 (85.3%/14.7%)	654/1057 (38.2%/61.8%)	< 0.001*
BMI, kg/m^2^	20.81 (18.69–23.33)	22.04 (19.99–24.31)	19.71 (17.98–22.10)	< 0.001*
CC, cm	32.00 (29.70–34.50)	33.00 (31.00–35.50)	31.00 (29.00–33.50)	< 0.001*
HGS, kg	24.56 (18.00–31.50)	26.00 (19.60–33.20)	23.50 (16.70–30.50)	< 0.001*
Albumin, g/L	38.20 (34.40–41.70)	38.90 (34.90–42.20)	37.50 (34.00–41.10)	< 0.001*
WBC, ×10^9^g/L	5.90 (4.60–7.85)	5.97 (4.72–7.90)	5.83 (4.46–7.82)	0.023*
Neutrophil, ×10^9^g/L	3.66 (2.60–5.44)	3.69 (2.70–5.30)	3.65 (2.52–5.53)	0.730
Lymphocyte, ×10^9^g/L	1.42 (1.05–1.84)	1.50 (1.10–1.90)	1.37 (1.00–1.80)	< 0.001*
Platelet, ×10^9^g/L	221.00 (167.00–285.00)	220.00 (167.00–279.00)	223.00 (169.00–289.00)	0.350
NLR	2.56 (1.63–4.37)	2.45 (1.60–4.06)	2.68 (1.66–4.70)	0.003*

Abbreviations: BMI, body mass index; CC, calf circumference; CHD, coronary heart disease; CONUT, controlling nutritional status; ECOG, Eastern Cooperative Oncology Group; HGS, hand grip strength; NLR, neutrophil to lymphocyte ratio; NRS2002, Nutrition Risk Screen 2002; PG‐SGA, Patient‐Generated Subjective Global Assessment; TNM, tumour/node/metastasis; WBC, white blood cell.

* *p* value < 0.05 was defined as statistical significance.

### 3.2 Comparison of EORTC QLQ‐C30 Questionnaire Between Cachexia Patients and Nonachexia Patients

The summary score of the EORTC QLQ‐C30 questionnaire for patients with gastric cancer and cachexia was 87.18 (IQR = 76.92–93.91), significantly lower than that of noncachexia patients, with a median value of 90.34 (IQR = 82.31–95.51) (Figure S2). In patients with cachexia, the scores for functional scales and the global quality of life scale were lower than the corresponding scores for patients without cachexia. Conversely, the scores of symptom scales were higher in patients with cachexia than in those without. Significant differences in patients with cachexia compared with those without were observed in most EORTC QLQ‐C30 scale scores except the emotional and cognitive function scales. More detailed information on the comparison is provided in Table 2.

**TABLE 2:** Comparison of QLQ‐C30 between gastric cancer patients with cachexia and without cachexia.

Variables	All patients (*n* = 3158) (median [IQR])	Without cachexia (*n* = 1447) (median [IQR])	With cachexia (*n* = 1711) (median [IQR])	*p*
Physical function	86.67 (73.33–100.00)	93.33 (80.00–100.00)	86.67 (66.67–100.00)	< 0.001*
Role function	83.33 (66.67–100.00)	100.00 (66.67–100.00)	83.33 (66.67–100.00)	< 0.001*
Emotional function	91.67 (75.00–100.00)	91.67 (75.00–100.00)	91.67 (75.00–100.00)	0.157
Cognitive function	100.00 (83.33–100.00)	100.00 (83.33–100.00)	100.00 (83.33–100.00)	0.069
Social function	66.67 (66.67–100.00)	66.67 (66.67–100.00)	66.67 (66.67–100.00)	< 0.001*
Global quality of life	66.67 (50.00–66.67)	66.67 (50.00–75.00)	58.33 (50.00–66.67)	< 0.001*
Fatigue	11.11 (0.00–33.33)	11.11 (0.00–33.33)	22.22 (0.00–33.33)	< 0.001*
Nausea and vomiting	0.00 (0.00–16.67)	0.00 (0.00–0.00)	0.00 (0.00–16.67)	< 0.001*
Pain	0.00 (0.00–16.67)	0.00 (0.00–16.67)	0.00 (0.00–33.33)	0.011*
Dyspnoea	0.00 (0.00–0.00)	0.00 (0.00–0.00)	0.00 (0.00–0.00)	< 0.001*
Insomnia	0.00 (0.00–33.33)	0.00 (0.00–33.33)	0.00 (0.00–33.33)	< 0.001*
Loss of appetite	0.00 (0.00–33.33)	0.00 (0.00–33.33)	0.00 (0.00–33.33)	< 0.001*
Constipation	0.00 (0.00–0.00)	0.00 (0.00–0.00)	0.00 (0.00–0.00)	< 0.001*
Diarrhoea	0.00 (0.00–0.00)	0.00 (0.00–0.00)	0.00 (0.00–0.00)	0.004*
Financial impact	33.33 (0.00–58.33)	33.33 (0.00–33.33)	33.33 (0.00–66.67)	0.004*
Summary score	88.72 (79.74–94.87)	90.34 (82.31–95.51)	87.18 (76.92–93.91)	< 0.001*

Abbreviation: IQR, interquartile range.

### 3.3 Association of Cachexia With Scales of EORTC QLQ‐C30

Using univariate logistic regression analysis to examine the association between baseline characteristics and cachexia, several baseline factors, including gender, TNM stage, surgery, radiotherapy, chemotherapy, ECOG grade, HGS and CC, were found to be significant (Table S1). Table 3 lists the significant scales in the EORTC QLQ‐C30 questionnaire associated with cachexia based on univariate logistic regression analyses. In multivariate logistic regression analyses adjusting for the significant factors in baseline characteristics above, physical function (OR = 1.43, 95% CI = 1.22–1.68, *p* < 0.001), global quality of life (OR = 1.22, 95% CI = 1.03–1.45, *p* = 0.022), fatigue (OR = 1.37, 95% CI = 1.17–1.60, *p* < 0.001), nausea and vomiting (OR = 1.82, 95% CI = 1.53–2.16, *p* < 0.001), dyspnoea (OR = 1.33, 95% = 1.10–1.62, *p* = 0.004), insomnia (OR = 1.23, 95% = 1.06–1.43, *p* = 0.007), loss of appetite (OR = 1.65, 95% CI = 1.42–1.93, *p* < 0.001), constipation (OR = 1.55, 95% CI = 1.28–1.88, *p* < 0.001), diarrhoea (OR = 1.33, 95% CI = 1.05–1.67, *p* = 0.017) and summary score (OR = 1.20, 95% CI = 1.10–1.42, *p* = 0.039) were identified as independent scales significantly associated with cachexia. As indicated by the time‐independent ROC curve, the loss of appetite scale had the largest AUC value of 0.570, followed by physical function, nausea and vomiting, fatigue, summary score, constipation, insomnia, global quality of life, dyspnoea and diarrhoea (Figure S3).

**TABLE 3:** Logistic regression analyses of QLQ‐C30 questionnaire scales for cachexia in gastric cancer patients.

	No. of patients	Crude model		Adjusted model^a^		Adjusted model^b^	
Variables		OR (95% CI)	*p*	OR (95% CI)	*p*	OR (95% CI)	*p*
Physical function							
= 100	1118	Reference					
< 100	2040	1.78 (1.53–2.06)	< 0.001*	1.73 (1.50–2.01)	< 0.001*	1.43 (1.22–1.68)	< 0.001*
Role function							
= 100	1475	Reference					
< 100	1683	1.39 (1.21–1.60)	< 0.001*	1.35 (1.17–1.56)	< 0.001*	1.09 (0.94–1.28)	0.249
Emotional function							
= 100	1516	Reference					
< 100	1642	1.05 (0.91–1.21)	0.503				
Cognitive function							
= 100	1772	Reference					
< 100	1386	1.12 (0.97–1.28)	0.130				
Social function							
= 100	1039	Reference					
< 100	2119	1.23 (1.06–1.43)	0.006*	1.20 (1.03–1.39)	0.018*	1.01 (0.86–1.18)	0.939
Global quality of life							
≥ 66.67	787	Reference					
< 66.67	2371	1.45 (1.23–1.70)	< 0.001*	1.42 (1.20–1.67)	< 0.001*	1.22 (1.03–1.45)	0.022*
Fatigue							
= 0	1111	Reference					
0	2047	1.57 (1.36–1.82)	< 0.001*	1.54 (1.33–1.79)	< 0.001*	1.37 (1.17–1.60)	< 0.001*
Nausea and vomiting							
= 0	2355	Reference					
0	803	1.96 (1.66–2.31)	< 0.001*	1.91 (1.61–2.26)	< 0.001*	1.82 (1.53–2.16)	< 0.001*
Pain							
= 0	1726	Reference					
0	1432	1.13 (0.98–1.30)	0.083				
Dyspnea							
= 0	2584	Reference					
0	574	1.47 (1.22–1.77)	< 0.001*	1.47 (1.22–1.77)	< 0.001*	1.33 (1.10–1.62)	0.004*
Insomnia							
= 0	1904	Reference					
0	1254	1.35 (1.17–1.56)	< 0.001*	1.34 (1.16–1.55)	< 0.001*	1.23 (1.06–1.43)	0.007*
Loss of appetite							
= 0	2016	Reference					
0	1142	1.86 (1.60–2.16)	< 0.001*	1.83 (1.57–2.13)	< 0.001*	1.65 (1.42–1.93)	< 0.001*
Constipation							
= 0	2584	Reference					
0	574	1.67 (1.38–2.01)	< 0.001*	1.66 (1.38–2.00)	< 0.001*	1.55 (1.28–1.88)	< 0.001*
Diarrhoea							
= 0	2793	Reference					
0	365	1.38 (1.10–1.72)	0.005*	1.38 (1.10–1.73)	0.005*	1.33 (1.05–1.67)	0.017*
Financial impact							
= 0	1011	Reference					
0	2147	1.21 (1.04–1.41)	0.012*	1.19 (1.02–1.38)	0.023*	1.10 (0.94–1.28)	0.234
Summary score							
≥ 94.87	812	Reference					
< 94.87	2346	1.48 (1.26–1.74)	< 0.001*	1.44 (1.23–1.70)	< 0.001*	1.20 (1.01–1.42)	0.039*

Abbreviations: CI, confidence interval; OR, odds ratio; SD, standard deviation.

^a^ Adjusted for age, gender and TNM stage.

^b^ Adjusted for variables found significant at *p* < 0.05 in the univariate analyses of baseline characteristics, including gender, TNM stage, surgery, radiotherapy, chemotherapy, ECOG grade, HGS and CC.

* *p* value < 0.05 was defined as statistical significance.

### 3.4 Association of Scales of EORTC QLQ‐C30 With Survival Outcome

**FIGURE 1:**
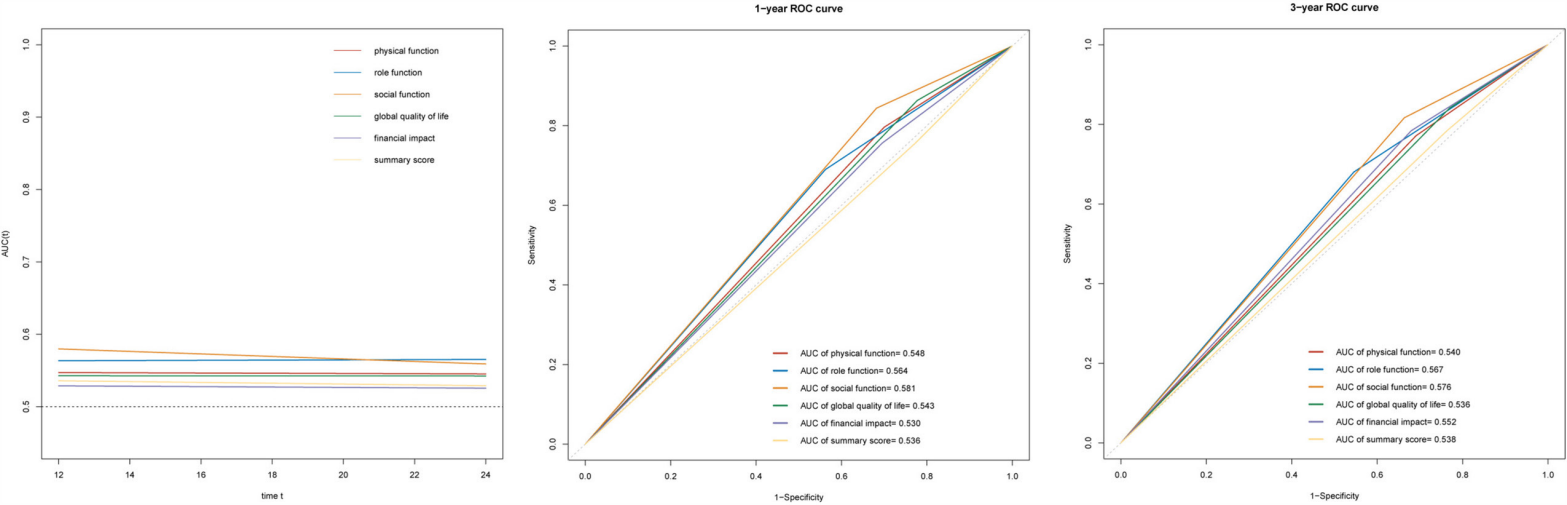
Comparison of ROC curves among independent predictors for survival in gastric cancer patients with cachexia, including physical function, role function, social function, global quality of life, financial impact and summary score.

**FIGURE 2:**
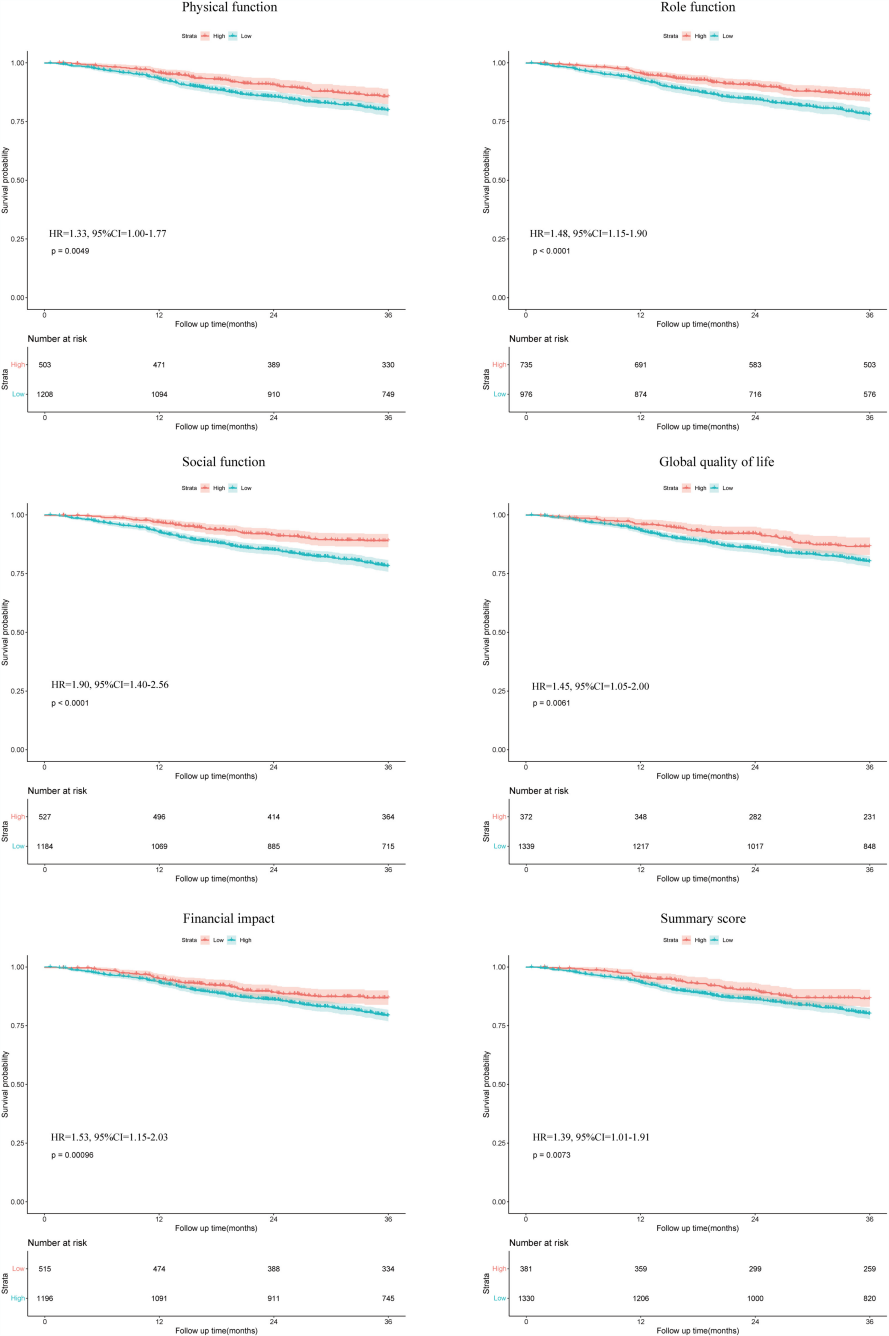
Kaplan–Meier curves for OS stratified by independent scales, including physical function, role function, social function, global quality of life, financial impact and summary score in gastric cancer patients with cachexia.

A total of 1711 patients with cachexia were included in the survival analysis. During the follow‐up period, 644 deaths occurred. Table S2 presents several significant baseline characteristics using univariate Cox proportional hazard regression analyses, including diabetes, hypertension, TNM stages, surgery, chemotherapy and HGS. Table 4 outlines scales with statistical significance in patients with gastric cancer and cachexia based on univariate Cox proportional hazard regression. In the multivariate Cox regression adjusting for significant baseline factors, physical function (HR = 1.33, 95% CI = 1.00–1.77, *p* = 0.049), role function (HR = 1.48, 95% CI = 1.15–1.90, *p* = 0.002), social function (HR = 1.90, 95% CI = 1.40–2.56, *p* < 0.001), global quality of life (HR = 1.45, 95% CI = 1.05–2.00, *p* = 0.026), financial impact (HR = 1.53, 95% CI = 1.15–2.03, *p* = 0.003) and summary score (HR = 1.39, 95% CI = 1.01–1.91, *p* = 0.042) were all independent risk factors associated with poor survival (Table 4). The C‐index of social function was 0.567 (95% CI = 0.543–0.590), role function was 0.561 (95% CI = 0.534–0.588), financial impact was 0.542 (95% CI = 0.517–0.567), physical function was 0.539 (95% CI = 0.515–0.564), global quality of life was 0.534 (95% CI = 0.513–0.555), and summary score was 0.533 (95% CI = 0.511–0.555) (Table S3). According to the time‐independent ROC curve, the AUC value for the social and role function scales consistently exceeded that of other independent risk scales associated with poor survival (Figure 1). The C‐index and AUC values for the social function were the highest, indicating greater accuracy in predicting survival compared with other scales. Additionally, Kaplan–Meier curves revealed that patients with high scores in the functional scales (physical function, role function and social function), global quality of life, or summary score, or low scores in the symptom scales (financial impact) demonstrated significantly longer survival times (Figure 2).

**TABLE 4:** Cox proportional hazard regression analyses of QLQ‐C30 questionnaire scales for OS in gastric cancer patients with cachexia.

Variables	No. of patients	Crude model		Adjusted model^a^		Adjusted model^b^	
		HR (95% CI)	*p*	HR (95% CI)	*p*	HR (95% CI)	*p*
Physical function							
= 100	503	Reference					
< 100	1208	1.48 (1.12–1.95)	0.005*	1.46 (1.10–1.92)	0.008*	1.33 (1.00–1.77)	0.049*
Role function							
= 100	735	Reference					
< 100	976	1.67 (1.31–2.14)	< 0.001*	1.59 (1.25–2.04)	< 0.001*	1.48 (1.15–1.90)	0.002*
Emotional function							
= 100	812	Reference					
< 100	899	1.06 (0.84–1.33)	0.638				
Cognitive function							
= 100	939	Reference					
< 100	772	1.14 (0.91–1.44)	0.256				
Social function							
= 100	527	Reference					
< 100	1184	2.09 (1.55–2.81)	< 0.001*	1.99 (1.48–2.68)	< 0.001*	1.90 (1.40–2.56)	< 0.001*
Global quality of life							
≥ 75	372	Reference					
< 75	1339	1.56 (1.13–2.15)	0.007*	1.51 (1.09–2.08)	0.012*	1.45 (1.05–2.00)	0.026*
Fatigue							
= 0	521	Reference					
0	1190	1.32 (1.01–1.72)	0.042*	1.30 (0.99–1.60)	0.057	1.21 (0.92–1.58)	0.175
Nausea and vomiting							
= 0	1179	Reference					
0	532	1.16 (0.91–1.48)	0.236				
Pain							
= 0	911	Reference					
0	800	0.95 (0.76–1.20)	0.669				
Dyspnoea							
= 0	1356	Reference					
0	355	1.07 (0.81–1.41)	0.655				
Insomnia							
= 0	975	Reference					
0	736	1.16 (0.92–1.46)	0.217				
Loss of appetite							
= 0	982	Reference					
0	729	1.06 (0.83–1.33)	0.668				
Constipation							
= 0	1342	Reference					
0	369	1.07 (0.82–1.41)	0.615				
Diarrhoea							
= 0	1488	Reference					
0	223	1.05 (0.75–1.47)	0.765				
Financial impact							
= 0	515	Reference					
0	1196	1.60 (1.21–2.12)	0.001*	1.56 (1.18–2.06)	0.002*	1.53 (1.15–2.03)	0.003*
Summary score							
≥ 94.87	381	Reference					
< 94.87	1330	1.53 (1.12–2.09)	0.008*	1.50 (1.10–2.05)	0.011*	1.39 (1.01–1.91)	0.042*

Abbreviations: CI, confidence interval; OR, odds ratio; SD, standard deviation.

^a^ Adjusted for age, gender and TNM stage.

^b^ Adjusted for variables found significant at *p* < 0.05 in the univariate analyses, including chemotherapy, diabetes, HGS, hypertension, surgery and TNM stages.

* *p* value < 0.05 was defined as statistical significance.

### 3.5 Results of Noncachexia Patients With Gastric Cancer

A total of 1447 patients without cachexia were enrolled in this study. During the follow‐up period, 520 deaths occurred. Table S4 shows the factors with statistical significance in baseline characteristics identified by univariate Cox proportional hazard regression analyses. After multivariate Cox proportional hazard regression analyses, adjusting for factors with statistical significance in baseline characteristics including CC, chemotherapy, NRS 2002, surgery and TNM stage, the scale identified as an independent predictor of survival in noncachexia patients with gastric cancer was the role function scale (HR = 1.39, 95% CI = 1.07–1.82, *p* = 0.015) (Table S5). The C‐index for this scale was 0.566 (95% CI = 0.535–0.596) (Table S6). According to the Kaplan–Meier curve in Figure S4, a high role function score was associated with prolonged survival in noncachexia patients with gastric cancer.

## 4 Discussion

Gastric cancer represents a significant public health challenge, characterized by a substantial burden of morbidity and mortality [19]. It is often diagnosed at an advanced stage and has a high incidence of cachexia [5]. In this study, the proportion of patients with gastric cancer diagnosed with cachexia was relatively large, especially among those in advanced stages, whose proportion was up to 56.7%. Cancer cachexia significantly impairs the QoL and exacerbates the symptom burden [20]. A recent study compared the QoL between patients with cachexia and those without, employing the EORTC QLQ‐C30 questionnaire. It determined that patients with cachexia exhibited significantly lower physical, role, cognitive, emotional, social functions, and overall QoL compared with those without cachexia [21]. In line with previous research, our study demonstrated that patients with cachexia exhibit lower scores on the functional scales and higher scores on the symptom scales compared with those without cachexia. However, both cachectic and noncachectic patients with gastric cancer in this study exhibited relatively high EORTC QLQ‐C30 summary scores. This may be partly attributed to the inclusion of early‐stage gastric cancer patients, who generally report better QoL due to a milder symptom burden and preserved functional status.

While numerous studies on cachexia have utilized the EORTC QLQ‐C30, most of them employed it to assess treatment efficacy, with QoL typically serving as a secondary endpoint. Limited research has explored the correlation between EORTC QLQ‐C30 scales and cancer cachexia. In our study, physical function, global quality of life, fatigue, nausea and vomiting, dyspnoea, insomnia, loss of appetite, constipation, diarrhoea and summary score were identified as independent scales associated with cachexia. This suggests that patients with cachexia are more likely to experience these dysfunctions and symptoms. Among them, loss of appetite exhibited the strongest association according to ROC analyses. Throughout disease progression, tumour‐bearing patients frequently experience reduced appetite due to both the malignancy itself and its treatment [22]. Survey data indicate that approximately 60% of patients with advanced cancer experience nausea and vomiting at various stages of their disease [23], which further exacerbate appetite loss [24]. These symptoms can contribute to inadequate nutrient intake, eventually resulting in malnutrition and cachexia, thus negatively impacting patients' QoL and clinical prognosis [25]. Fatigue is another common and debilitating symptom in cancer patients. As hallmark features of cachexia, skeletal muscle wasting and malnutrition can deplete energy reserves and contribute to persistent fatigue [26, 27]. Additionally, muscle atrophy contributes to a significant decline in strength, limiting the patient's ability to engage in physical activities, such as walking and daily self‐care [28]. Fatigue and muscle weakness conjointly compromise patients' physical function. Attention to these symptoms in clinical practice and scientific inquiry could aid in managing the onset and progression of cachexia.

Numerous studies have demonstrated that cancer cachexia is frequently associated with diminished survival [6, 29]. In line with previous studies, this study identified physical function, role function, social function, global quality of life, financial impact and summary scores as independent predictors of survival in patients with gastric cancer and cachexia. Among these, the social function scale demonstrated the highest accuracy based on the C‐index and ROC analyses. The social function scale predominantly assesses patients' engagement in social activities and their capacity to fulfil social roles. Individuals with cachexia frequently experience weight loss, muscle atrophy, anxiety and depression, and diminished energy, which may limit their capacity to engage in social interactions [30]. Similarly, the role function scale, which evaluates the impact of health on work, family responsibilities and leisure activities, was significantly associated with survival outcomes. This finding aligns with numerous previous studies demonstrating a close association between role function and all‐cause mortality in patients with cancer [31]. Moreover, financial strain emerged as a significant survival predictor. Individuals of higher socioeconomic status generally have greater access to healthcare services, healthier lifestyles and broader health knowledge, all of which contribute to improved survival outcomes [32].

Additionally, we investigated the impact of EORTC QLQ‐C30 scales on the survival of patients with gastric cancer without cachexia. We discovered that only the role function scale is independently associated with survival in these patients, a finding that is less pronounced than in patients with gastric cancer who have cachexia. For this result, we hypothesized that because patients with gastric cancer without cachexia are often in the early stages of cancer, the QoL is not significantly affected, and the impacts on function and symptoms are less pronounced. Consequently, the predictive value of the EORTC QLQ‐C30 for survival in these patients is limited.

Our study has some limitations. First, the data included in our survey were available only in Chinese since they were sourced from medical institutions in China. Therefore, the association between EORTC QLQ‐C30 scales and cachexia, and survival in patients with gastric cancer warrants further investigation in more diverse populations, encompassing various ethnicities. Second, potential unmeasured confounders may have been overlooked, potentially influencing our findings.

## 5 Conclusion

In summary, our study has identified key scales within the EORTC QLQ‐C30 significantly affected by cachexia in patients with gastric cancer and has also established specific scales as independent predictors of survival in patients with gastric cancer who have cachexia. Concentrating on these symptoms and functional scales of the EORTC QLQ‐C30 could assist clinicians in evaluating the impact of cachexia in patients with gastric cancer and in forecasting their survival outcomes.

## Ethics Statement

In accordance with the principles of the Declaration of Helsinki, ethical approval for this study was obtained from the Medical Ethics Committee of the First Affiliated Hospital of Sun Yat‐sen University (reference number 2013‐82); each participating institution also provided ethical approval. Patients were enrolled after being fully informed of the study's purpose and provided signed informed consent.

## Conflicts of Interest

The authors declare no conflicts of interest.

## Supporting information


**Table S1.** Logistic regression analyses of baseline characteristics for cachexia in gastric cancer patients. Abbreviation: TNM, tumour/node/metastasis; NRS2002, Nutrition Risk Screen 2002; ECOG, Eastern Cooperative Oncology Group; BMI, body mass index; HGS, hand grip strength; CC, calf circumference. *P value < 0.05 was defined as statistical significance.
**Table S2.** Cox regression analyses of baseline characteristics for OS in gastric cancer patients with cachexia. Abbreviation: TNM, tumour/node/metastasis; NRS2002, Nutrition Risk Screen 2002; ECOG, Eastern Cooperative Oncology Group; BMI, body mass index; HGS, hand grip strength; CC, calf circumference. *P value < 0.05 was defined as statistical significance.
**Table S3.** C‐index of independent scales, including physical function, role function, social function, global quality of life, financial impact, and summary score for survival prediction in gastric cancer patients with cachexia. Abbreviations: C‐index, Concordance index
**Table S4.** Cox regression analyses of baseline characteristics for OS in gastric cancer patients without cachexia. Abbreviation: TNM, tumour/node/metastasis; NRS2002, Nutrition Risk Screen 2002; ECOG, Eastern Cooperative Oncology Group; BMI, body mass index; HGS, hand grip strength; CC, calf circumference. *P value < 0.05 was defined as statistical significance.
**Table S5.** Cox proportional hazard regression analyses of QLQ‐C30 questionnaire scales for OS in gastric cancer patients without cachexia. Abbreviation: SD, standard deviation; OR, odds ratio; CI, confidence interval. ^a^ Adjusted for age, gender, TNM stages.^b^ Adjusted for variables found significant at *p* < 0.05 in the univariate analyses, including CC, chemotherapy, NRS 2002, surgery, TNM stage.* P value < 0.05 was defined as statistical significance.
**Table S6.** C‐index of independent scale named role function for survival prediction in gastric cancer patients without cachexia. Abbreviations: C‐index, Concordance index
**Figure S1.** Flow chart.
**Figure S2.** Comparison of summary scores of QLQ‐C30 between gastric cancer patients with and without cachexia.
**Figure S3.** Comparison of ROC curves among independent scales associated with cachexia in gastric cancer patients, including physical function, global quality of life, fatigue, nausea and vomiting, dyspnea, insomnia, loss of appetite, constipation, diarrhoea, and summary score. AUC, area under curve; ROC, receiver operating characteristic.
**Figure S4.** Kaplan–Meier curves for OS stratified by independent scales, including role function in gastric cancer patients without cachexia.

## Data Availability

The datasets that were created and analysed for this study are accessible upon request from the corresponding author, provided the request is reasonable and justifiable.
