# Supplementary material for: A Comprehensive Analysis of the Association Between the EORTC QLQ‐C30 Questionnaire and Cachexia in Patients With Gastric Cancer
Source: J Cachexia Sarcopenia Muscle. 2025 Jun 12;16(3):e13859. doi: 10.1002/jcsm.13859 (PMC12160612; doi:10.1002/jcsm.13859)

**TABLES**

**TABLE S1** Logistic regression analyses of baseline characteristics for cachexia in gastric cancer patients

| **Variables** | **No. of patients** | **Univariate** | |  | **Multivariate** | |
| --- | --- | --- | --- | --- | --- | --- |
|  |  | **OR (95%CI)** | **P value** |  | **OR (95%CI)** | **P value** |
| Age, years |  |  |  |  |  |  |
| <65 | 1323 | Reference |  |  |  |  |
| ≥65 | 1835 | 0.99 (0.86-1.14) | 0.873 |  |  |  |
| Gender |  |  |  |  |  |  |
| Male | 2178 | Reference |  |  |  |  |
| Female | 980 | 1.19 (1.02-1.38) | 0.025* |  | 1.01 (0.86-1.18) | 0.928 |
| Smoking |  |  |  |  |  |  |
| No | 1742 | Reference |  |  |  |  |
| Yes | 1416 | 0.93 (0.81-1.08) | 0.344 |  |  |  |
| Drinking |  |  |  |  |  |  |
| No | 2459 | Reference |  |  |  |  |
| Yes | 699 | 0.97 (0.82-1.15) | 0.749 |  |  |  |
| Diabetes |  |  |  |  |  |  |
| No | 2945 | Reference |  |  |  |  |
| Yes | 213 | 0.86 (0.65-1.14) | 0.292 |  |  |  |
| Hypertension |  |  |  |  |  |  |
| No | 2667 | Reference |  |  |  |  |
| Yes | 491 | 1.06 (0.87-1.29) | 0.556 |  |  |  |
| TNM stages |  |  |  |  |  |  |
| Ⅰ/Ⅱ | 1088 | Reference |  |  |  |  |
| Ⅲ/Ⅳ | 2070 | 1.34 (1.16-1.56) | <0.001* |  | 1.18 (1.01-1.38) | 0.038* |
| Surgery |  |  |  |  |  |  |
| No | 1693 | Reference |  |  |  |  |
| Yes | 1465 | 0.63 (0.55-0.73) | <0.001* |  | 0.85 (0.68-1.06) | 0.158 |
| Radiotherapy |  |  |  |  |  |  |
| No | 3069 | Reference |  |  |  |  |
| Yes | 89 | 3.00 (1.81-4.96) | <0.001* |  | 2.51 (1.50-4.22) | <0.001* |
| Chemotherapy |  |  |  |  |  |  |
| No | 1859 | Reference |  |  |  |  |
| Yes | 1299 | 1.46 (1.26-1.68) | <0.001* |  | 1.26 (1.00-1.58) | 0.046* |
| ECOG grade |  |  |  |  |  |  |
| ≤1 | 2594 | Reference |  |  |  |  |
| >1 | 564 | 1.61 (1.33-1.94) | <0.001* |  | 1.58 (1.30-1.92) | <0.001* |
| HGS, kg |  |  |  |  |  |  |
| High (≥35.6) | 477 | Reference |  |  |  |  |
| Low (<35.6) | 2681 | 1.70 (1.40-2.07) | <0.001* |  | 1.37 (1.11-1.69) | 0.004* |
| CC, cm |  |  |  |  |  |  |
| High (≥36) | 498 | Reference |  |  |  |  |
| Low (<36) | 2660 | 2.60 (2.13-3.18) | <0.001* |  | 2.25 (1.83-2.77) | <0.001* |

Abbreviation: TNM, tumor/node/metastasis; NRS2002, Nutrition Risk Screen 2002; ECOG, Eastern Cooperative Oncology Group; BMI, body mass index; HGS, hand grip strength; CC, calf circumference.

*P value<0.05 was defined as statistical significance.

**TABLE S2** Cox regression analyses of baseline characteristics for OS in gastric cancer patients with cachexia

| **Variables** | **No. of patients** | **Univariate** | |  | **Multivariate** | |
| --- | --- | --- | --- | --- | --- | --- |
|  |  | **HR (95%CI)** | **P value** |  | **HR (95%CI)** | **P value** |
| Age, years |  |  |  |  |  |  |
| <65 | 719 | Reference |  |  |  |  |
| ≥65 | 992 | 1.11 (0.88-1.40) | 0.394 |  |  |  |
| Gender |  |  |  |  |  |  |
| Male | 1151 | Reference |  |  |  |  |
| Female | 560 | 1.03 (0.80-1.31) | 0.842 |  |  |  |
| Smoking |  |  |  |  |  |  |
| No | 957 | Reference |  |  |  |  |
| Yes | 754 | 0.92 (0.73-1.16) | 0.467 |  |  |  |
| Drinking |  |  |  |  |  |  |
| No | 1336 | Reference |  |  |  |  |
| Yes | 375 | 0.98 (0.74-1.30) | 0.881 |  |  |  |
| Diabetes |  |  |  |  |  |  |
| No | 1603 | Reference |  |  |  |  |
| Yes | 108 | 1.79 (1.22-2.62) | 0.003* |  | 1.69 (1.14-2.51) | 0.009* |
| Hypertension |  |  |  |  |  |  |
| No | 1439 | Reference |  |  |  |  |
| Yes | 272 | 1.48 (1.12-1.96) | 0.006* |  | 1.44 (1.08-1.92) | 0.014* |
| TNM stages |  |  |  |  |  |  |
| Ⅰ/Ⅱ | 537 | Reference |  |  |  |  |
| Ⅲ/Ⅳ | 1174 | 3.54 (2.51-5.00) | <0.001* |  | 3.50 (2.47-4.96) | 0.001* |
| Surgery |  |  |  |  |  |  |
| No | 1006 | Reference |  |  |  |  |
| Yes | 705 | 0.67 (0.53-0.86) | 0.001* |  | 0.97 (0.68-1.38) | 0.851 |
| Radiotherapy |  |  |  |  |  |  |
| No | 1642 | Reference |  |  |  |  |
| Yes | 69 | 1.32 (0.79-2.22) | 0.292 |  |  |  |
| Chemotherapy |  |  |  |  |  |  |
| No | 936 | Reference |  |  |  |  |
| Yes | 775 | 1.47 (1.17-1.86) | 0.001* |  | 1.25 (0.89-1.74) | 0.199 |
| NRS2002 |  |  |  |  |  |  |
| <3 | 183 | Reference |  |  |  |  |
| ≥3 | 1528 | 1.39 (0.91-2.10) | 0.126 |  |  |  |
| ECOG grade |  |  |  |  |  |  |
| ≤1 | 1352 | Reference |  |  |  |  |
| >1 | 359 | 1.20 (0.91-1.56) | 0.193 |  |  |  |
| BMI, kg/m^2^ |  |  |  |  |  |  |
| High (≥18.5) | 1151 | Reference |  |  |  |  |
| Low (<18.5) | 560 | 1.03 (0.81-1.31) | 0.813 |  |  |  |
| HGS, kg |  |  |  |  |  |  |
| High (≥35.6) | 205 | Reference |  |  |  |  |
| Low (<35.6) | 1506 | 1.67 (1.09-2.55) | 0.019* |  | 1.72 (1.12-2.63) | 0.013* |
| CC, cm |  |  |  |  |  |  |
| High (≥36) | 172 | Reference |  |  |  |  |
| Low (<36) | 1539 | 1.20 (0.80-1.81) | 0.387 |  |  |  |

Abbreviation: TNM, tumor/node/metastasis; NRS2002, Nutrition Risk Screen 2002; ECOG, Eastern Cooperative Oncology Group; BMI, body mass index; HGS, hand grip strength; CC, calf circumference.

*P value<0.05 was defined as statistical significance.

**TABLE S3** C-index of independent scales, including physical function, role function, social function, global quality of life, financial impact, and summary score for survival prediction in gastric cancer patients with cachexia

| **Variables** | **C-index** | **95% Confidence interval** |
| --- | --- | --- |
| Physical function | 0.539 | 0.515-0.564 |
| Role function | 0.561 | 0.534-0.588 |
| Social function | 0.567 | 0.543-0.590 |
| Global quality of life | 0.534 | 0.513-0.555 |
| Financial impact | 0.542 | 0.517-0.567 |
| Summary score | 0.533 | 0.511-0.555 |

Abbreviations: C-index, Concordance index

**TABLE S4** Cox regression analyses of baseline characteristics for OS in gastric cancer patients without cachexia

| **Variables** | **No. of patients** | **Univariate** | |  | **Multivariate** | |
| --- | --- | --- | --- | --- | --- | --- |
|  |  | **HR (95%CI)** | **P value** |  | **HR (95%CI)** | **P value** |
| Age, years |  |  |  |  |  |  |
| <65 | 604 | Reference |  |  |  |  |
| ≥65 | 843 | 1.18 (0.91-1.52) | 0.218 |  |  |  |
| Gender |  |  |  |  |  |  |
| Male | 1027 | Reference |  |  |  |  |
| Female | 420 | 0.76 (0.57-1.02) | 0.065 |  |  |  |
| Smoking |  |  |  |  |  |  |
| No | 785 | Reference |  |  |  |  |
| Yes | 662 | 1.05 (0.82-1.34) | 0.715 |  |  |  |
| Drinking |  |  |  |  |  |  |
| No | 1123 | Reference |  |  |  |  |
| Yes | 324 | 1.08 (0.81-1.45) | 0.609 |  |  |  |
| Diabetes |  |  |  |  |  |  |
| No | 1342 | Reference |  |  |  |  |
| Yes | 105 | 1.26 (0.82-1.96) | 0.296 |  |  |  |
| Hypertension |  |  |  |  |  |  |
| No | 1228 | Reference |  |  |  |  |
| Yes | 219 | 1.05 (0.74-1.48) | 0.789 |  |  |  |
| TNM stages |  |  |  |  |  |  |
| Ⅰ/Ⅱ | 551 | Reference |  |  |  |  |
| Ⅲ/Ⅳ | 896 | 3.64 (2.59-5.10) | <0.001* |  | 3.29 (2.32-4.67) | 0.001* |
| Surgery |  |  |  |  |  |  |
| No | 687 | Reference |  |  |  |  |
| Yes | 760 | 0.63 (0.49-0.81) | <0.001* |  | 0.98 (0.66-1.47) | 0.937 |
| Radiotherapy |  |  |  |  |  |  |
| No | 1427 | Reference |  |  |  |  |
| Yes | 20 | 0.26 (0.04-1.82) | 0.174 |  |  |  |
| Chemotherapy |  |  |  |  |  |  |
| No | 923 | Reference |  |  |  |  |
| Yes | 524 | 1.69 (1.32-2.17) | <0.001* |  | 1.24 (0.84-1.84) | 0.287 |
| NRS2002 |  |  |  |  |  |  |
| <3 | 800 | Reference |  |  |  |  |
| ≥3 | 647 | 1.56 (1.22-2.00) | <0.001* |  | 1.39 (1.08-1.79) | 0.012* |
| ECOG grade |  |  |  |  |  |  |
| ≤1 | 1242 | Reference |  |  |  |  |
| >1 | 205 | 1.22 (0.88-1.70) | 0.242 |  |  |  |
| BMI, kg/m^2^ |  |  |  |  |  |  |
| High (≥18.5) | 1272 | Reference |  |  |  |  |
| Low (<18.5) | 175 | 1.31 (0.93-1.85) | 0.128 |  |  |  |
| HGS, kg |  |  |  |  |  |  |
| High (≥35.6) | 272 | Reference |  |  |  |  |
| Low (<35.6) | 1175 | 1.07 (0.77-1.49) | 0.676 |  |  |  |
| CC, cm |  |  |  |  |  |  |
| High (≥36) | 326 | Reference |  |  |  |  |
| Low (<36) | 1121 | 1.58 (1.13-2.22) | 0.008* |  | 1.41 (1.00-2.00) | 0.049* |

Abbreviation: TNM, tumor/node/metastasis; NRS2002, Nutrition Risk Screen 2002; ECOG, Eastern Cooperative Oncology Group; BMI, body mass index; HGS, hand grip strength; CC, calf circumference.

*P value<0.05 was defined as statistical significance.

**TABLE S5** Cox proportional hazard regression analyses of QLQ-C30 questionnaire scales for OS in gastric cancer patients without cachexia.

| **Variables** | **No. of patients** | **Crude model** | |  | **Adjusted model ^a^** | |  | **Adjusted model ^b^** | |
| --- | --- | --- | --- | --- | --- | --- | --- | --- | --- |
|  |  | **HR (95%CI)** | **P value** |  | **HR (95%CI)** | **P value** |  | **HR (95%CI)** | **P value** |
| Physical function |  |  |  |  |  |  |  |  |  |
| **=**100 | 615 | Reference |  |  |  |  |  |  |  |
| <100 | 832 | 1.24 (0.96-1.61) | 0.100 |  |  |  |  |  |  |
| Role function |  |  |  |  |  |  |  |  |  |
| **=**100 | 740 | Reference |  |  |  |  |  |  |  |
| <100 | 707 | 1.67 (1.31-2.17) | <0.001* |  | 1.58 (1.22-2.04) | <0.001* |  | 1.39 (1.07-1.82) | 0.015* |
| Emotional function |  |  |  |  |  |  |  |  |  |
| **=**100 | 704 | Reference |  |  |  |  |  |  |  |
| <100 | 734 | 1.03 (0.80-1.32) | 0.823 |  |  |  |  |  |  |
| Cognitive function |  |  |  |  |  |  |  |  |  |
| **=**100 | 833 | Reference |  |  |  |  |  |  |  |
| <100 | 614 | 0.85 (0.66-1.09) | 0.203 |  |  |  |  |  |  |
| Social function |  |  |  |  |  |  |  |  |  |
| **=**100 | 512 | Reference |  |  |  |  |  |  |  |
| <100 | 935 | 1.27 (0.97-1.66) | 0.086 |  |  |  |  |  |  |
| Global quality of life |  |  |  |  |  |  |  |  |  |
| ≥75 | 415 | Reference |  |  |  |  |  |  |  |
| <75 | 1032 | 1.32 (0.98-1.76) | 0.066 |  |  |  |  |  |  |
| Fatigue |  |  |  |  |  |  |  |  |  |
| **=**0 | 590 | Reference |  |  |  |  |  |  |  |
| >0 | 857 | 1.22 (0.94-1.58) | 0.129 |  |  |  |  |  |  |
| Nausea and vomiting |  |  |  |  |  |  |  |  |  |
| **=**0 | 1176 | Reference |  |  |  |  |  |  |  |
| >0 | 271 | 1.18 (0.87-1.60) | 0.297 |  |  |  |  |  |  |
| Pain |  |  |  |  |  |  |  |  |  |
| **=**0 | 815 | Reference |  |  |  |  |  |  |  |
| >0 | 632 | 0.82 (0.64-1.06) | 0.126 |  |  |  |  |  |  |
| Dyspnea |  |  |  |  |  |  |  |  |  |
| **=**0 | 1228 | Reference |  |  |  |  |  |  |  |
| >0 | 219 | 0.88 (0.61-1.26) | 0.471 |  |  |  |  |  |  |
| Insomnia |  |  |  |  |  |  |  |  |  |
| **=**0 | 929 | Reference |  |  |  |  |  |  |  |
| >0 | 518 | 1.23 (0.95-1.59) | 0.111 |  |  |  |  |  |  |
| Loss of appetite |  |  |  |  |  |  |  |  |  |
| **=**0 | 1034 | Reference |  |  |  |  |  |  |  |
| >0 | 413 | 1.27 (0.98-1.66) | 0.074 |  |  |  |  |  |  |
| Constipation |  |  |  |  |  |  |  |  |  |
| **=**0 | 1242 | Reference |  |  |  |  |  |  |  |
| >0 | 205 | 0.99 (0.69-1.42) | 0.960 |  |  |  |  |  |  |
| Diarrhea |  |  |  |  |  |  |  |  |  |
| **=**0 | 1305 | Reference |  |  |  |  |  |  |  |
| >0 | 142 | 0.69 (0.43-1.12) | 0.129 |  |  |  |  |  |  |
| Financial impact |  |  |  |  |  |  |  |  |  |
| **=**0 | 496 | Reference |  |  |  |  |  |  |  |
| >0 | 951 | 1.07 (0.82-1.40) | 0.606 |  |  |  |  |  |  |
| Summary score |  |  |  |  |  |  |  |  |  |
| ≥94.87 | 431 | Reference |  |  |  |  |  |  |  |
| <94.87 | 1016 | 1.44 (1.07-1.93) | 0.015* |  | 1.33 (0.99-1.79) | 0.062 |  | 1.14 (0.84-1.55) | 0.396 |

Abbreviation: SD, standard deviation; OR, odds ratio; CI, confidence interval.

^a^ Adjusted for age, gender, TNM stages.

^b^ Adjusted for variables found significant at p<0.05 in the univariate analyses, including CC, chemotherapy, NRS 2002, surgery, TNM stage.

^*^ P value<0.05 was defined as statistical significance.

**TABLE S6** C-index of independent scale named role function for survival prediction in gastric cancer patients without cachexia

| **Variables** | **C-index** | **95% Confidence interval** |
| --- | --- | --- |
| Role function | 0.566 | 0.535-0.596 |

Abbreviations: C-index, Concordance index

**FIGURES**

**FIGURE S1** Flow chart


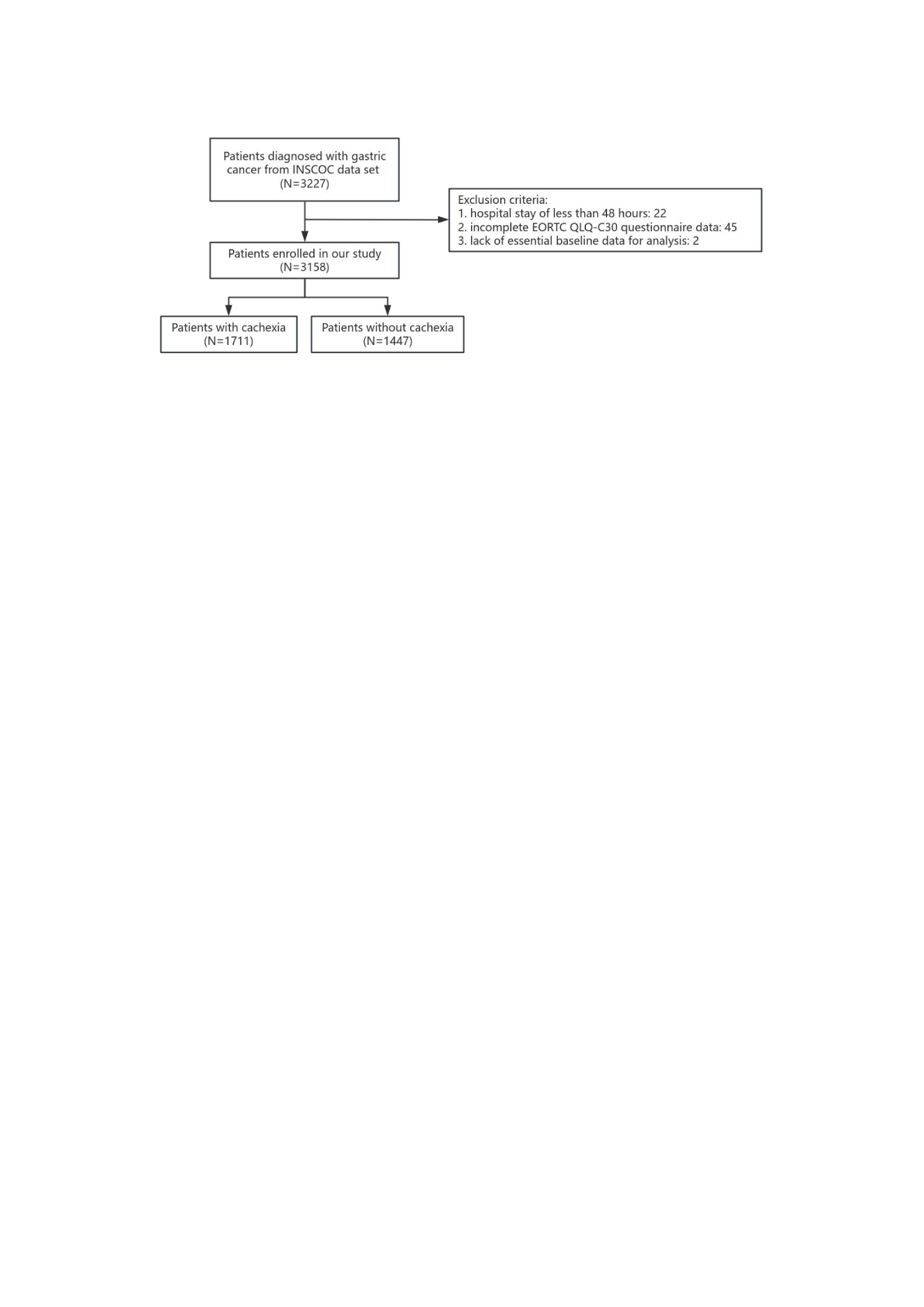


**FIGURE S2** Comparison of summary scores of QLQ-C30 between gastric cancer patients with and without cachexia


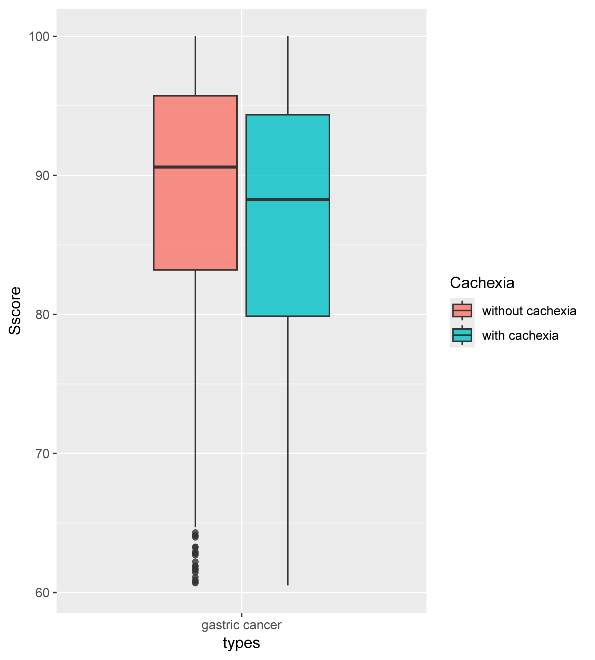


**FIGURE S3** Comparison of ROC curves among independent scales associated with cachexia in gastric cancer patients, including physical function, global quality of life, fatigue, nausea and vomiting, dyspnea, insomnia, loss of appetite, constipation, diarrhea, and summary score.


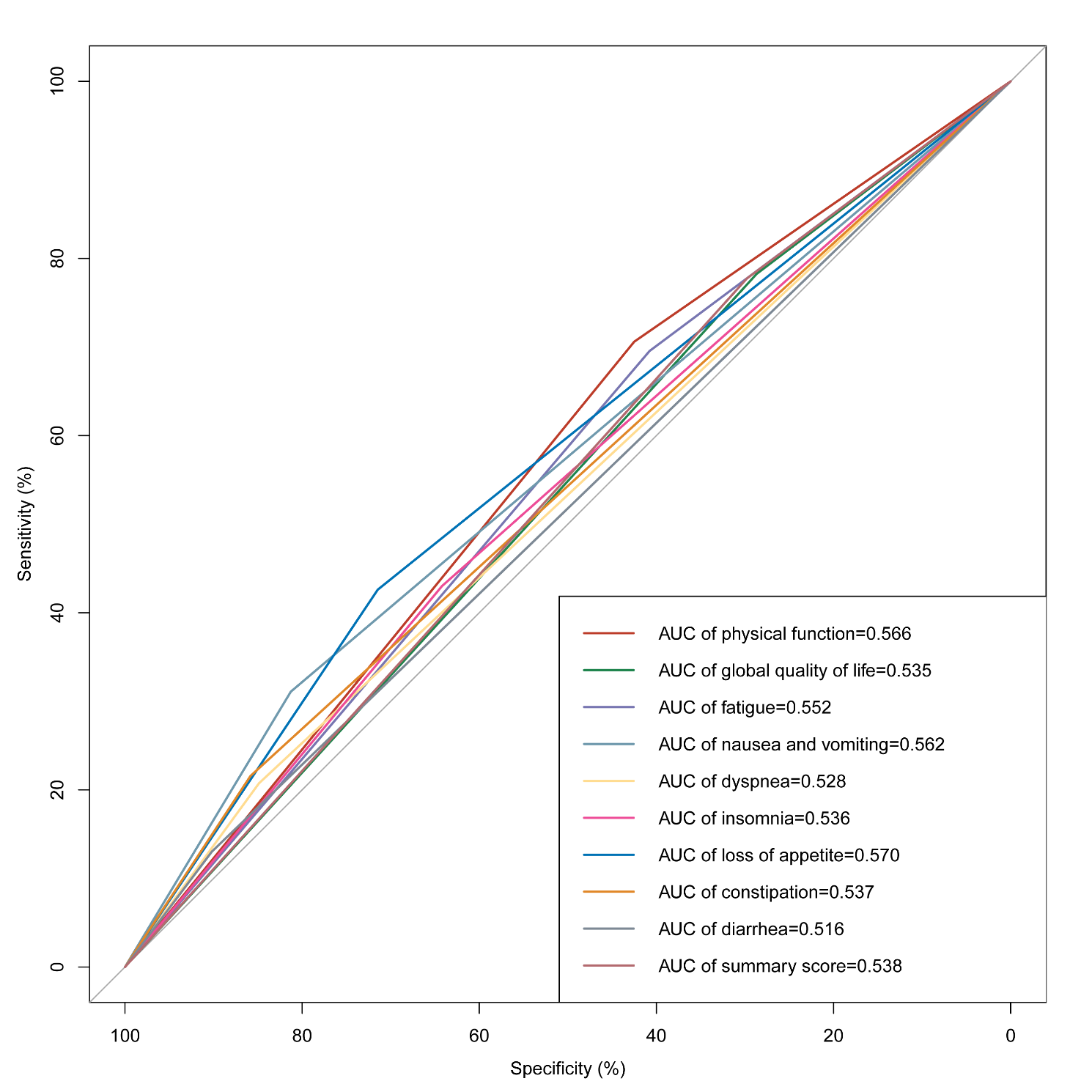


AUC, area under curve; ROC, receiver operating characteristic.

**FIGURE S4** Kaplan-Meier curves for OS stratified by independent scales, including role function in gastric cancer patients without cachexia


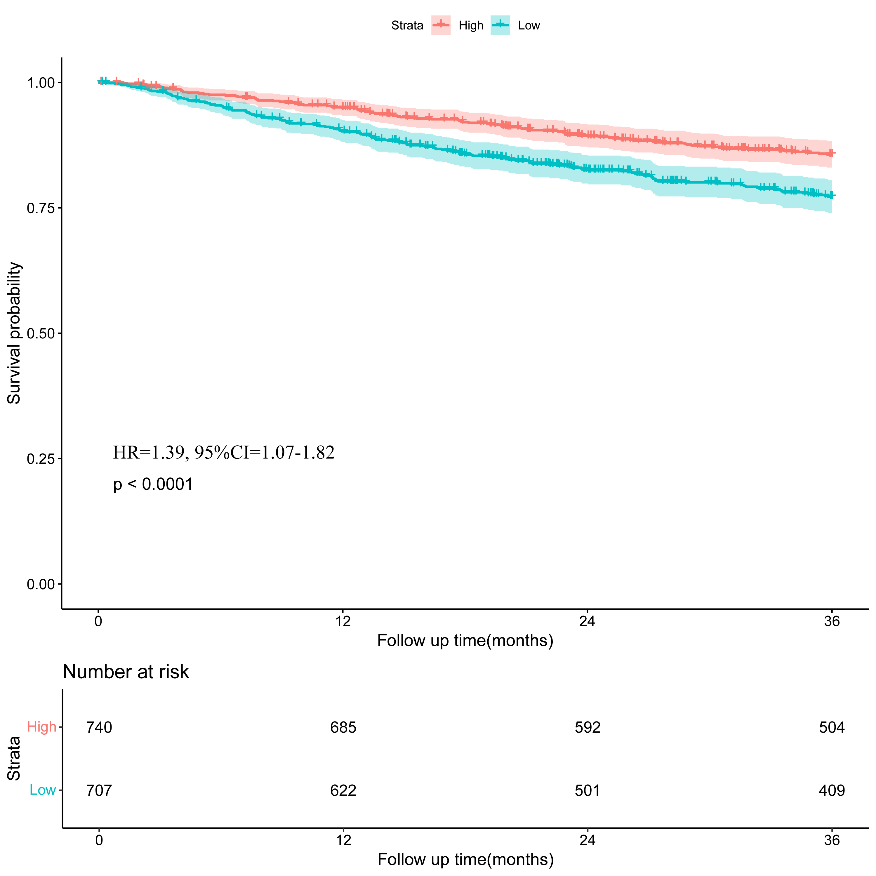

Supplement: Supplementary file 1 — Table S1. Logistic regression analyses of baseline characteristics for cachexia in gastric cancer patients. Abbreviation: TNM, tumour/node/metastasis; NRS2002, Nutrition Risk Screen 2002; ECOG, Eastern Cooperative Oncology Group; BMI, body mass index; HGS, hand grip strength; CC, calf circumference. *P value < 0.05 was defined as statistical significance. Table S2. Cox regression analyses of baseline characteristics for OS in gastric cancer patients with cachexia. Abbreviation: TNM, tumour/node/metastasis; NRS2002, Nutrition Risk Screen 2002; ECOG, Eastern Cooperative Oncology Group; BMI, body mass index; HGS, hand grip strength; CC, calf circumference. *P value < 0.05 was defined as statistical significance. Table S3. C‐index of independent scales, including physical function, role function, social function, global quality of life, financial impact, and summary score for survival prediction in gastric cancer patients with cachexia. Abbreviations: C‐index, Concordance index Table S4. Cox regression analyses of baseline characteristics for OS in gastric cancer patients without cachexia. Abbreviation: TNM, tumour/node/metastasis; NRS2002, Nutrition Risk Screen 2002; ECOG, Eastern Cooperative Oncology Group; BMI, body mass index; HGS, hand grip strength; CC, calf circumference. *P value < 0.05 was defined as statistical significance. Table S5. Cox proportional hazard regression analyses of QLQ‐C30 questionnaire scales for OS in gastric cancer patients without cachexia. Abbreviation: SD, standard deviation; OR, odds ratio; CI, confidence interval. a Adjusted for age, gender, TNM stages.b Adjusted for variables found significant at p < 0.05 in the univariate analyses, including CC, chemotherapy, NRS 2002, surgery, TNM stage.* P value < 0.05 was defined as statistical significance. Table S6. C‐index of independent scale named role function for survival prediction in gastric cancer patients without cachexia. Abbreviations: C‐index, Concordance index Figure S1. Flow chart. [file JCSM-16-e13859-s001.docx]
